# Oxidant Exposure Induces Cysteine-Rich Protein 61 (CCN1) via c-Jun/AP-1 to Reduce Collagen Expression in Human Dermal Fibroblasts

**DOI:** 10.1371/journal.pone.0115402

**Published:** 2014-12-23

**Authors:** Zhaoping Qin, Patrick Robichaud, Tianyuan He, Gary J. Fisher, John J. Voorhees, Taihao Quan

**Affiliations:** Department of Dermatology, University of Michigan Medical School, Ann Arbor, Michigan, United States of America; The University of Hong Kong, Hong Kong

## Abstract

Human skin is a primary target of oxidative stress from reactive oxygen species (ROS) generated from both extrinsic and intrinsic sources. Oxidative stress inhibits the production of collagen, the most abundant protein in skin, and thus contributes to connective tissue aging. Here we report that cysteine-rich protein 61 (CCN1), a negative regulator of collagen production, is markedly induced by ROS and mediates loss of type I collagen in human dermal fibroblasts. Conversely, antioxidant N-acetyl-L-cysteine significantly reduced CCN1 expression and prevented ROS-induced loss of type I collagen in both human dermal fibroblasts and human skin *in vivo*. ROS increased c-Jun, a critical member of transcription factor AP-1 complex, and increased c-Jun binding to the AP-1 site of the CCN1 promoter. Functional blocking of c-Jun significantly reduced CCN1 promoter and gene expression and thus prevented ROS-induced loss of type I collagen. Targeting the c-Jun/CCN1 axis may provide clinical benefit for connective tissue aging in human skin.

## Introduction

Oxidative stress is an important pathogenic factor involved in human aging [1], [2]. Human skin is exposed to reactive oxygen species (ROS) generated from both environmental sources like ultraviolet irradiation (UV) (photoaging) [3], [4] and endogenous oxidative metabolism (natural aging) [5]. These oxidative stresses impair the synthesis of collagen, the major structural protein in skin, and potentiate skin connective tissue aging [5], [6]. A characteristic feature of aged human skin is thinning of the dermis and diminished tensile strength caused by loss of dermal connective tissue collagen [7]–[9]. Loss of collagen has deleterious effects on skin structural integrity and function, and contributes to common aged-related skin problems such as impaired wound healing and increased risk for skin cancer [10]–[13].

In human skin, dermal fibroblasts are responsible for the production of collagen and other extra cellular matrix (ECM) proteins. We previously reported that cysteine-rich protein 61 (CCN1), a secreted and extracellular matrix-associated protein, is predominantly expressed in human skin dermal fibroblasts, and is substantially elevated in the dermis of naturally aged [14], [15], photoaged [14], [16] and acutely UV-irradiated human skin *in vivo* [17], [18]. Importantly, elevated CCN1 functions as a negative regulator of collagen production [14], [15], [17].

CCN1 is a member of the CCN protein family, which consists of six distinct members; cysteine-rich protein 61 (CCN1); connective tissue growth factor (CCN2); nephroblastoma overexpressed (CCN3); Wnt-inducted secreted protein-1 (CCN4); Wnt-inducted secreted protein-2 (CCN5); and Wnt-inducted secreted protein-3 (CCN6) [19]–[21]. CCN family proteins exhibit diverse cellular functions including regulation of cell adhesion, proliferation, migration, chemotaxis, apoptosis, motility, and ECM remodeling [19], [20], [22].

ROS is considered an important driving force in the aging process and elevated CCN1 in aged human skin functions as a novel mediator of human skin aging. Presently, we investigated regulation of CCN1 expression by ROS and the role of CCN1 in ROS-induced loss of type I collagen. Our data demonstrate that ROS up-regulates CCN1 through transcription factor AP-1, and that elevated CCN1 mediates loss of type I collagen caused by ROS in human skin dermal fibroblasts.

## Results

### Oxidant exposure induces CCN1 expression and inhibits type I procollagen

**Figure 1:**
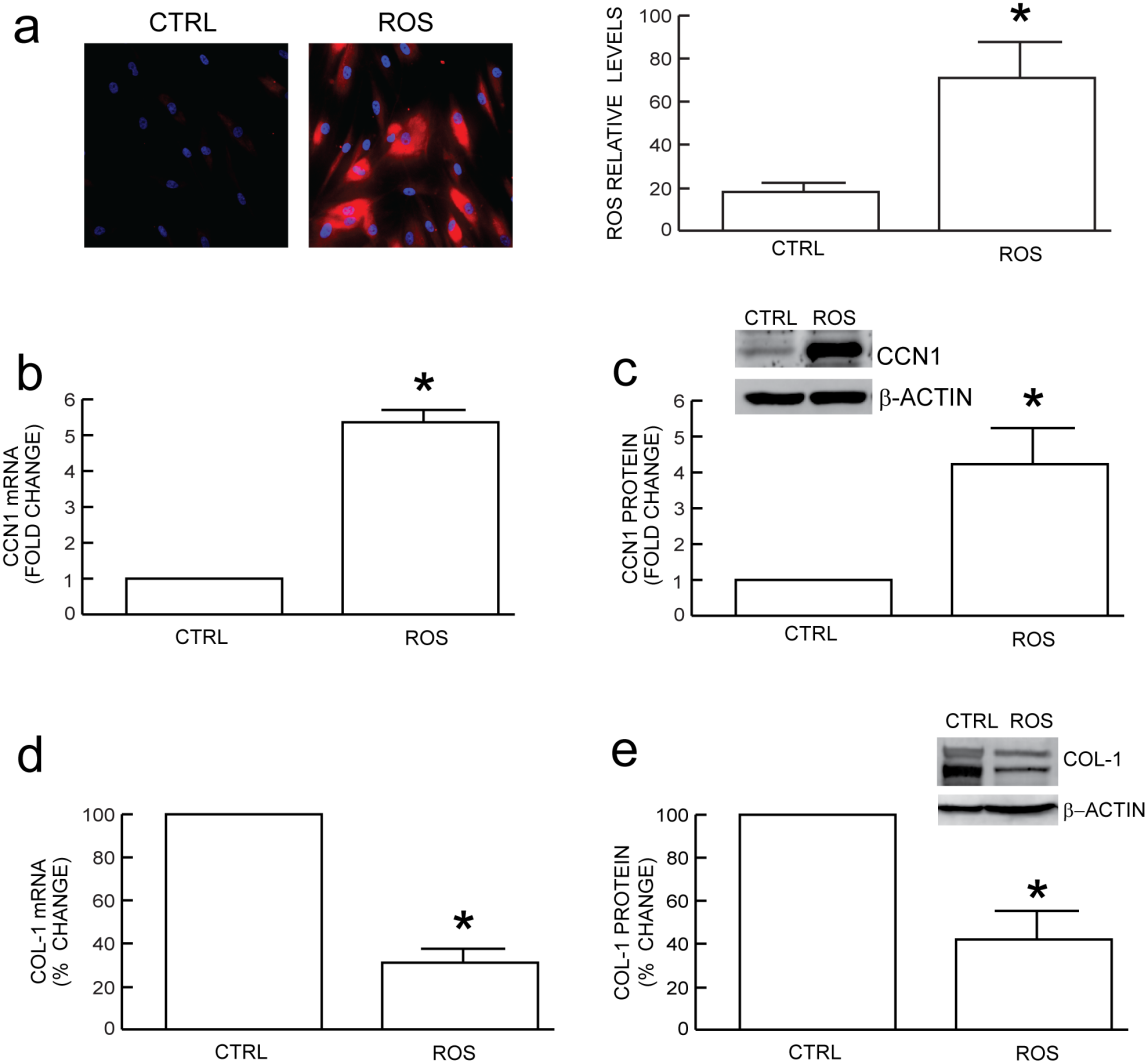
Oxidative exposure induces CCN1 expression and inhibits type I procollagen in adult human primary dermal fibroblasts. Fibroblasts were exposed to vehicle (CTRL) or H_2_O_2_ on two successive days (ROS), as described in Methods. (a) Oxidative exposure induces endogenous ROS. RedoxSensor Red CC-1, an indicator of ROS level, was visualized and quantified by fluorescence microscopy, as described in Methods. Oxidative exposure induces CCN1 (b) mRNA and (c) protein. Oxidative exposure reduces Type I procollagen (COL-1) (d) mRNA and (e) protein. mRNA levels were quantified by real-time RT-PCR and normalized to an internal control housekeeping gene (36B4). Protein levels were determined by Western blot and normalized to an internal control (β-actin). Inset shows representative blot. Data are means±SEM, N = 3, *p<0.05 vs. CTRL.

We first investigated the response of human dermal fibroblasts to low levels of the naturally occurring pro-oxidant hydrogen peroxide. Cultured dermal fibroblasts were exposed to low dose hydrogen peroxide for one hour on two successive days (see Materials and Methods for details). The cells were extensively washed after each exposure to remove the added hydrogen peroxide and placed in fresh DMEM. We found that this short-term exposure (one hour) of dermal fibroblasts to low dose hydrogen peroxide (150 µM) on two successive days caused long-lasting elevation of intracellular ROS levels. Elevated intracellular ROS levels did not diminish for at least 21 days (the longest time we examined). We used the cells seven days after removing added hydrogen peroxide, and refer to elevated intracellular ROS, as demonstrated in Fig. 1a, as ROS throughout the study. ROS increased CCN1 mRNA (Fig. 1b) and protein (Fig. 1c) and decreased type I procollagen (COL-1) mRNA (Fig. 1d) and protein (Fig. 1e). These data indicate that ROS up-regulates CCN1, a novel negative regulator of type I procollagen, as observed in naturally aged [14], [15], photoaged [14], [16] and UV-irradiated human skin [17], [18].

### CCN1 mediates ROS-induced loss of type I procollagen

**Figure 2:**
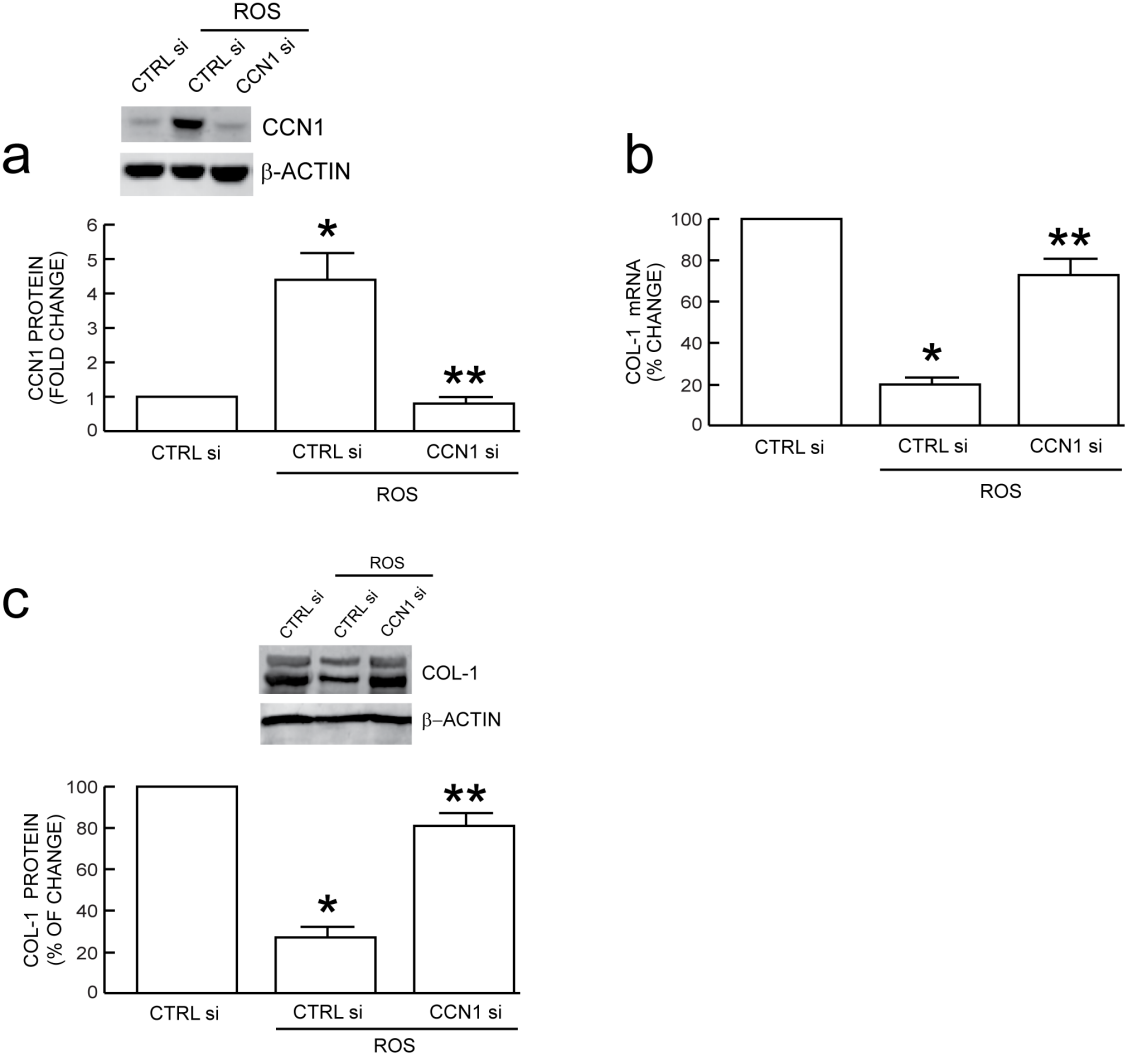
CCN1 induction by oxidative exposure mediates type I procollagen reduction in human dermal fibroblasts. Fibroblasts were exposed to vehicle (CTRL) or H_2_O_2_ on two successive days (ROS), as described in Methods. Cells were transfected with control siRNA (CTRL si) or CCN1 siRNA (CCN1 si), and harvested two days later. (a) CCN1 siRNA reduces oxidant-induced CCN1 protein. Knockdown of oxidant-induced CCN1 prevents reduction of type I procollagen (COL-1) (b) mRNA and (c) protein. mRNA levels were quantified by real-time RT-PCR normalized to internal control housekeeping gene (36B4). COL-1 protein levels were determined by Western blot normalized to an internal control (β-actin). Inset shows representative blot. Data are means±SEM, N = 3, *p<0.05 vs. CTRL, **p<0.05 vs. oxidative exposure with CTRL si.

We have previously reported that dermal fibroblasts, in aged human skin *in vivo*, express elevated levels of CCN1, and elevated CCN1 significantly inhibits type I collagen production [14], [16], [17], [23], [24]. These previous findings, coupled with ROS-mediated up-regulation of CCN1 expression described above, prompted us to investigate the effect of CCN1 knockdown in ROS-mediated reduction of type I collagen production. CCN1 siRNA markedly reduced CCN1 mRNA (S1a Figure) and protein (S1b Figure) levels. This reduction of CCN1 did not significantly alter the basal levels of type I procollagen mRNA expression in untreated control human dermal fibroblasts (S1c Figure). Importantly, knockdown of CCN1 significantly attenuated ROS-mediated reduction of type I procollagen mRNA (Fig. 2b) and protein (Fig. 2c). These data indicate that elevated CCN1 mediates, at least in part, loss of type I procollagen caused by ROS in human dermal fibroblasts.

### Antioxidant (NAC) treatment reduces ROS-mediated induction of CCN1 and loss of intracellular type I procollagen

**Figure 3:**
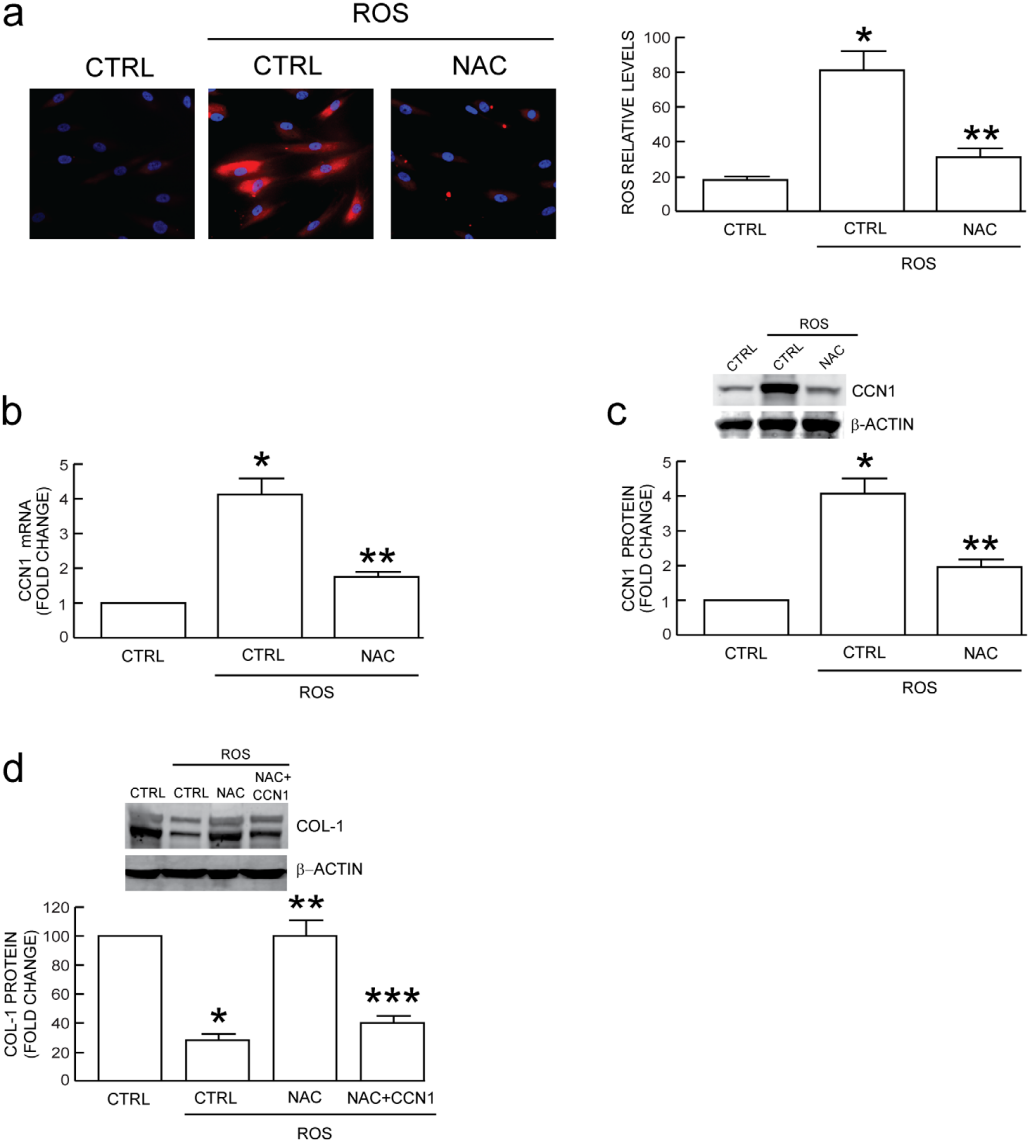
Antioxidant treatment reduces oxidant-induced CCN1 and loss of type I procollagen in human dermal fibroblasts. Fibroblasts were exposed to vehicle (CTRL) or H_2_O_2_ on two successive days (ROS), as described in Methods. Oxidant-exposed human dermal fibroblasts were treated with vehicle (CTRL) or N-acetylcysteine (NAC, 10 µM) for 24 hours. (a) NAC treatment prevents oxidant-induced ROS levels. Oxidation of RedoxSensor Red CC-1 was visualized and quantified by fluorescence microscopy. NAC treatment reduces oxidant-induced CCN1 (b) mRNA and (c) protein. (d) NAC or CCN1 expression attenuates loss of type I procollagen (COL-1) protein by oxidative exposure. mRNA levels were quantified by real-time RT-PCR normalized to an internal control housekeeping gene (36B4). Protein levels were determined by Western blot normalized to an internal control (β-actin). Inset shows representative blot. Data are means±SEM, N = 3, *p<0.05 vs CTRL, **p<0.05 vs CTRL with oxidative exposure, ***p<0.05 vs. NAC with oxidative exposure.

To investigate whether antioxidant treatment could attenuate CCN1 expression and prevent reduction of type I collagen, we treated dermal fibroblasts with N-acetyl-L-cysteine (NAC), a well-characterized antioxidant [25]. NAC treatment did not significantly alter the basal levels of CCN1 or type I procollagen expression in untreated control cells (S2 Figure). However, NAC markedly reduced ROS (Fig. 3a), CCN1 mRNA (Fig. 3b) and CCN1 protein levels (Fig. 3c), and consequently prevented ROS-mediated reduction of intracellular type I procollagen (Fig. 3d). This ability of NAC to preserve type I collagen was overcome by forced expression of CCN1 (Fig. 3d), consistent with our previous finding that elevated CCN1 inhibits type I collagen production [14], [16], [17], [23], [24]. These data demonstrated that antioxidant treatment attenuates ROS induction of CCN1 expression and largely prevents CCN1-mediated reduction of type I procollagen, in human dermal fibroblasts.

### ROS activates the CCN1 promoter, dependent upon a functional AP-1 binding site

**Figure 4:**
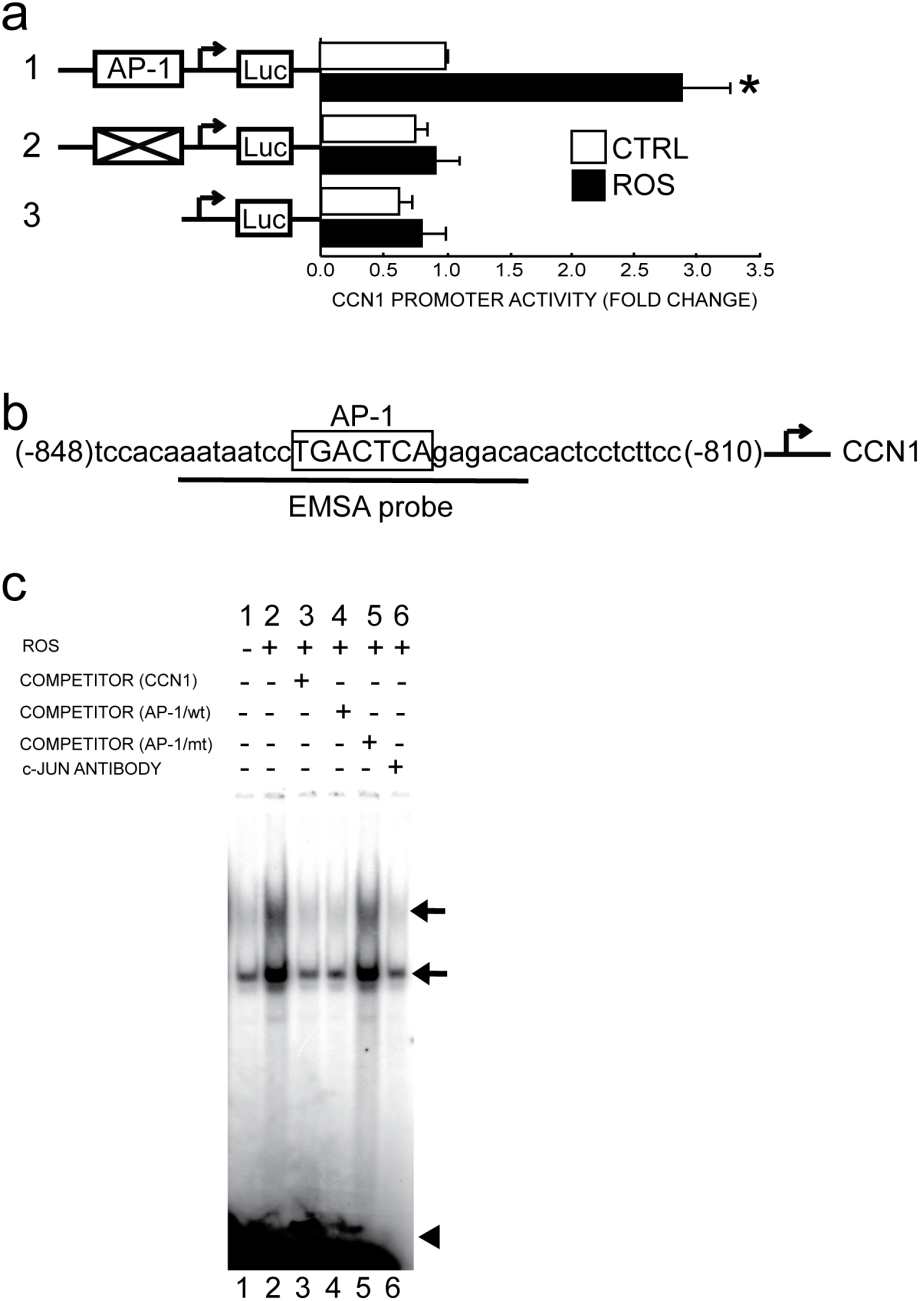
Oxidant exposure activates AP-1 binding site-dependent CCN1 promoter activity in human dermal fibroblasts. Fibroblasts were exposed to vehicle (CTRL) or H_2_O_2_ on two successive days (ROS), as described in Methods. Fibroblasts were transfected with CCN1 promoter/luciferase reporter constructs: 1, wild-type (−883/+1); 2, AP-1 mutation (−883/+1); and 3, AP-1 deletion (605/+1). Luciferase activities were determined two days after transfection. (a) Mutation or deletion of AP-1 binding site abolishes activation of CCN1 promoter by oxidative exposure. Data are means±SEM, N = 3, *p<0.05 vs. CTRL. (b) Nucleotide sequence of the CCN1 proximal promoter. AP-1-binding sequences are marked in box. Heavy underline denotes electrophoretic mobility shift assay (EMSA) probe. Arrow indicates transcription start site. (c) Oxidant-exposure increases protein-binding to AP-1 site in CCN1 promoter. DNA/protein complex was analyzed by EMSA. Arrows indicate specific retarded complexes. Closed triangle indicates free probes. Results are representative of at least three independent experiments.

Next, we investigated the molecular mechanisms by which ROS up-regulates CCN1 gene expression. Since CCN1 is regulated at a transcriptional level by various stimuli including UV irradiation [17], [26], [27], we first explored whether ROS stimulates the CCN1 promoter. In human dermal fibroblasts, a CCN1 promoter reporter construct (−883/+1, Fig. 4a, number 1 reporter) is activated ROS (3-fold). These data suggest that induction of CCN1 expression by oxidant exposure reflects activation of CCN1 promoter activity.

The CCN1 promoter contains a binding site (−834/−828) for transcription factor AP-1 which plays an important role in CCN1 promoter regulation by various stimuli [17], [27]. Therefore, we tested two reporter constructs: AP-1 binding site mutant construct (Fig. 4a, number 2 reporter); and AP-1 binding site deletion construct (Fig. 4a, number 3 reporter). Either AP-1 binding site mutation or deletion substantially abolished ROS-induced CCN1 promoter activity. These data indicate that stimulation of CCN1 promoter by ROS is dependent on the presence of an AP-1 binding site.

We next examined protein binding to the activator protein-1 (AP-1) element in the CCN1 promoter. Electrophoretic mobility shift assays (EMSA) were performed using labeled probe containing the AP-1 binding region (−842/−822) of the CCN1 promoter (Fig. 4b). With nuclear extracts from control dermal fibroblasts, two retarded complexes were observed (Fig. 4c, lane 1). ROS substantially increased the intensity of the retarded DNA/protein complexes (Fig. 4c, lane 2). ROS-induced DNA/protein complex formation was nearly abolished by excess unlabeled probe (Fig. 4c, lane 3) or unlabeled consensus AP-1 probe (Fig. 4c, lane 4), but not by mutant AP-1 probe (Fig. 4c, lane 5). Pre-incubation of nuclear extracts with antibody against c-Jun, a major component of AP-1 complex, but not by control IgG antibody (data not shown), markedly reduced the intensities of DNA/protein complex bands (Fig. 4c, lane 6). Taken together, these data demonstrate that CCN1 elevation following exposure to ROS results primarily from AP-1-dependent activation of CCN1 gene promoter, in human dermal fibroblasts.

### c-Jun is induced by oxidative stress, and functional blockage of c-Jun reduces ROS-induced CCN1 expression and prevents loss of type I procollagen

**Figure 5:**
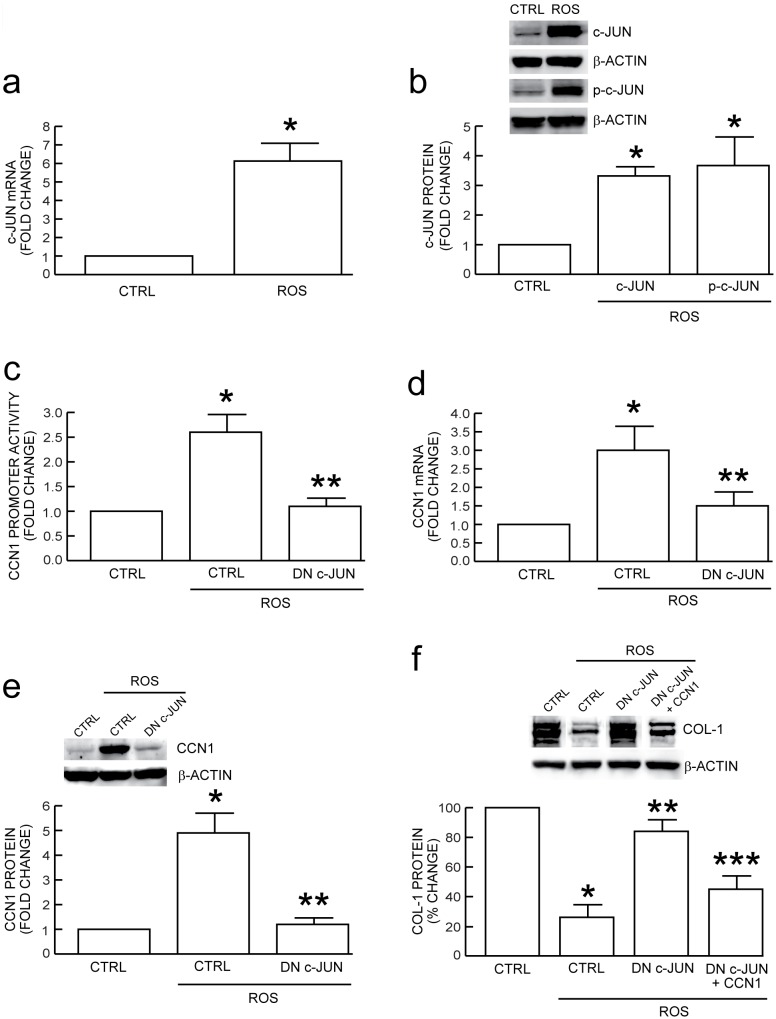
c-Jun mediates oxidative exposure induction of CCN1 and reduction of type I procollagen in human dermal fibroblasts. Fibroblasts were exposed to vehicle (CTRL) or H_2_O_2_ on two successive days (ROS), as described in Methods. (a) Oxidative exposure induces c-Jun mRNA and (b) total and phospho c-Jun proteins. mRNA levels were quantified by real-time RT-PCR normalized to an internal control housekeeping gene (36B4). Protein levels were determined by Western blot normalized to internal control (β-actin). Inset shows representative blot. (c) Dominant negative c-Jun (DN c-Jun) reduces oxidant activation of CCN1 promoter. Fibroblasts were co-transfected with CCN1 promoter/luciferase reporter construct (−883/+1) and control (CTRL) or DN c-Jun expression vector, and analyzed two days later. (d–f) DN c-Jun reduces oxidant induction of (d) CCN1 mRNA and (e) CCN1 protein. Fibroblasts were transfected with CTRL or DN c-Jun expression vectors, and analyzed two days later. (f) DN c-Jun mitigates loss of type I procollagen (COL-1) by oxidative exposure. Fibroblasts were transfected with CTRL or DN c-Jun alone or together with CCN1 expression vectors, and analyzed two days later. Data are means±SEM, N = 3, *p<0.05 vs. CTRL, **p<0.05 vs. CTRL with oxidative stress, ***p<0.05 vs DN-c-Jun alone.

Given that c-Jun is involved in AP-1 binding to the CCN1 promoter, we examined induction of c-Jun following oxidative stress. c-Jun mRNA (Fig. 5a) and c-Jun total and phosphorylated proteins (Fig. 5b) were markedly induced by ROS, consistent with elevated c-Jun positively regulating CCN1 gene expression in response to oxidant exposure. To further examine the role of c-Jun, we expressed dominant negative c-Jun (DN c-Jun, TAM67) that lacks transactivation domain [17], [28], [29]. Functional blocking of c-Jun resulted in significant inhibition of ROS-induced CCN1 promoter activity (Fig. 5c) and expression of CCN1 mRNA (Fig. 5d) and protein (Fig. 5e). Finally, we examined the impact of DN c-Jun on type I procollagen expression. DN c-Jun markedly prevented ROS-induced loss of type I procollagen, and this protection against collagen loss was significantly attenuated by CCN1 over-expression (Fig. 5f). These data demonstrate that c-Jun/AP-1 mediates ROS-induced CCN1 expression, and reducing CCN1 by DN c-Jun partially prevents loss of type I procollagen.

### Antioxidant treatment prevents elevation of c-Jun and CCN1 and protects against loss of type I collagen in UV-irradiated human skin in vivo

**Figure 6:**
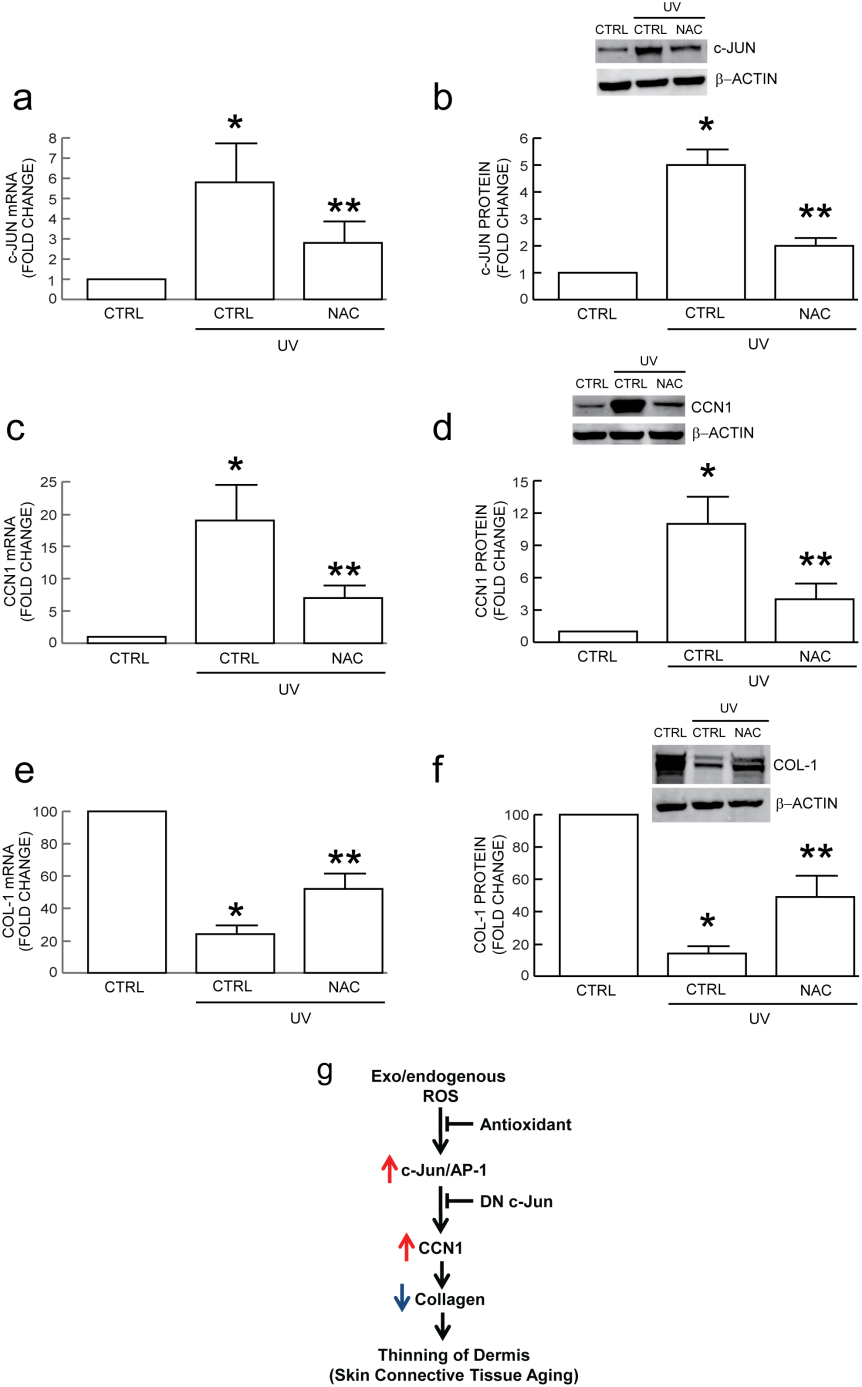
Topical antioxidant treatment blocks induction of c-Jun and CCN1 and loss of type I procollagen in UV-irradiated human skin *in vivo*. Human buttocks were treated topically with vehicle (CTRL) or N-acetylcysteine (NAC) 24 hours prior to UV irradiation (2MED). Skin samples were obtained eight hours after UV irradiation, as described in *Methods*. NAC attenuates UV irradiation induction of c-Jun (a) mRNA and (b) protein, and CCN1 (c) mRNA and (d) protein in human skin. NAC attenuates UV irradiation reduction of type I procollagen (e) mRNA and (f) protein in human skin. mRNA levels were quantified by real-time RT-PCR normalized to an internal control housekeeping gene (36B4). Protein levels were determined by Western blot normalized to an internal control (β-actin). Inset shows representative blot. Data are means±SEM, N = 6, *p<0.05 vs. CTRL no UV, **p<0.05 vs. CTRL + UV. (g) Working model for oxidant driven dermal aging through the c-Jun/CCN1 axis (see “Discussion” for details).

Solar UV irradiation generates ROS that mediate many of the harmful effects of UV radiation on skin. Therefore, we examined whether antioxidant treatment could prevent UV irradiation induction of c-Jun and CCN1 and protect against loss of type I collagen gene expression, in human skin *in vivo*. Punch biopsies were obtained from human skin treated topically with vehicle or NAC prior to exposure to two minimal erythema dose of solar simulated UV irradiation. UV irradiation induced c-Jun mRNA (Fig. 6a) and protein (Fig. 6b) 6-fold, and 6-fold, respectively. NAC reduced this induction by 60%. Similarly, UV irradiation induced CCN1 mRNA (Fig. 6c) and protein (Fig. 6d), and NAC reduced this induction by 65%. UV irradiation reduced type I collagen mRNA (Fig. 6e) and protein (Fig. 6f) 75%, and NAC attenuated this reduction by 50%. These data indicate that an antioxidant can efficiently reduce UV irradiation induction of c-Jun and CCN1 gene expression, and associated reduction of type I collagen mRNA, in human skin *in vivo*.

## Discussion

The oxidative stress theory of aging is a widely accepted hypothesis for the molecular basis of aging [1], [30]. Like other organs, human skin is exposed to ROS generated from aerobic metabolism (natural aging). In addition, human skin is a major target for a broad spectrum of external stressors, such as solar UV radiation and microbial and chemical assaults. These environmental factors directly or indirectly generate ROS that can lead to oxidative stress-mediated skin connective tissue injury.

The protective effects of antioxidants have been well-studied in cell culture systems and in animal models [31]. However, in humans, antioxidant based therapies have been generally disappointing [32]. Human skin is an excellent and accessible model organ to study the protective effects of antioxidants. Our data indicate that NAC, a well-known antioxidant, effectively prevents UV/ROS induction of c-Jun and its downstream effector CCN1. NAC is a metabolic precursor of glutathione, a co-factor for the enzyme glutathione peroxidase, which reduces hydrogen peroxide to water and is among the most abundant endogenous antioxidants. NAC is safe for human use, and we previously demonstrated that NAC penetrates human skin and effectively mitigates ROS-driven responses to acute UV in human skin *in vivo* [25].

Abnormal regulation of CCN1 protein expression has been observed in various diseases, including wound healing and fibrosis [33], [34]. Studies in a mouse model have demonstrated that CCN1 exerts potent anti-fibrotic activity via induction of dermal fibroblast senescence during cutaneous wound healing [35]. Replicative senescence is a form of cellular aging, and we reported that CCN1 mRNA and protein levels are significantly elevated in replicative senescent dermal fibroblasts [24]. Replicative senescent dermal fibroblasts express significantly reduced levels of type I procollagen and increased levels of MMP-1. Knockdown of elevated CCN1 in senescent dermal fibroblasts partially normalized both type I procollagen and MMP-1 expression, suggesting an important role of CCN1 in regulation of senescence-associated aberrant collagen homeostasis [9]. Interestingly, CCN1 is not only induced by oxidative exposure, but also increases intracellular ROS levels [35], [36], suggesting that a positive feedback loop involving CCN1 and ROS may cause sustained elevation of ROS and CCN1 in fibroblasts in aged human skin. Investigating this possibility is a worthwhile goal for future research. Secreted CCN1 may function as “docking” protein to facilitate interactions between cell surface receptors and ECM proteins. CCN1 has been shown to exert a range of diverse functions through interaction with multiple integrins in a cell-type, function-specific manner [37]. We reported that in human skin dermal fibroblasts CCN1 interacts with αVβ3 integrin to mediate up-regulation of MMP-1 [38]. Further investigation will be required to determine the role of integrin(s) in CCN1-dependent inhibition of type I collagen in response to ROS. CCN1 is the initial member of the CCN family of proteins, which also includes other five members. We investigated the effect of ROS on the other five CCN protein members. In contract to CCN1, ROS significantly reduced expression of CCN2 (S3 Figure). CCN1 and CCN2 are functionally distinct. CCN2 is well-known as a marker and mediator of tissue fibrosis [22], [37]. We have previously reported that CCN2 is a physiological regulator of collagen expression and reduced expression of CCN2 in aged human skin is involved in collagen loss in aged human skin [9]. These data suggest that ROS-mediated inhibition of collagen production involves not only elevated CCN1 expression but also reduced CCN2 expression. ROS also significantly reduced expression of CCN4 and CCN5 (S3 Figure). As the functions of CCN4 and CCN5 in collagen are unknown, further investigation will be required to determine the role of these proteins in ROS-mediated inhibition of type I collagen.

In addition to AP-1, functional binding motifs for other transcription factors, such as cAMP response element-binding protein (CREB), forkhead transcription factor (FOXO3), and early growth response-1 (EGR-1), have been identified in CCN1 proximal promoter region [26], [27], [39]. Our data (Fig. 4a) indicate that AP-1 functions as a major regulator of CCN1 in response to ROS, since deletion or mutation of AP-1 biding site alone, without altering any other transcription factors binding sites, is sufficient to completely eliminate ROS responsiveness. Furthermore, EMSA indicated that ROS induces protein-binding to the AP-1 response element in the CCN1 promoter. Two retarded complexes were observed. Both complexes contained c-Jun, a major component of AP-1 transcription factor, as shown by interference of complex formation by c-Jun antibody (Fig. 4c). c-Jun can homodimerize and heterodimerize with other AP-1 proteins, such as Fos family members [40], [41]. Although the precise molecular compositions of the two specific c-Jun-containing retarded complexes formed with the CCN1 promoter probe are not clear, it is conceivable that they contain distinct combinations of AP-1 proteins, and thus display different retarded mobility. c-Jun expression is largely regulated by its own gene product via phosphorylation by c-Jun NH2-terminal kinases (JNKs), a well-known stress-activated protein kinases [42]. The c-Jun promoter contains two AP-1 binding motifs, and thus c-Jun autoregulates its own promoter [43], [44]. As ROS act as potent activator of JNK [42], [45], [46], it is likely that ROS can increase c-Jun through activation of JNK. Indeed, Lo et al reported that ROS increases c-Jun expression via activation of JNK in chondrocytes [42]. AP-1 is a ubiquitous transcription factor that regulates multiple target genes involved in stress responses and cellular fate [47]. TGF-β pathway is the major regulator of ECM production in human dermal fibroblasts. We have previously reported that AP-1 induces Smad7, which blocks TGF-β signaling though inhibition of TGF-β type I receptor-dependent phosphorylation of Smad2/3, in human dermal fibroblasts [48]. Thus, AP-1 negatively regulates both components of the TGF-β receptor complex, albeit through distinct mechanisms. These data indicate that the balance between AP-1 and TGF-β pathways plays a crucial role in regulating ECM production in human dermal fibroblasts.

We have also reported that elevated CCN1 impairs TGF-β signaling through down-regulation of TGF-β type II receptor (TβRII) [14], suggesting elevated CCN1 inhibits collagen production by impairment of TGF-β signaling. Importantly, the TGF-β pathway is impaired in UV-irradiated, chronically photodamaged, and naturally aged human skin *in vivo*, largely due to down regulation of the TβRII [14], [49]. Our current results indicate that ROS promotes skin connective tissue aging through up-regulation of AP-1/CCN1 axis, as shown in Fig. 6d. Human skin is a primary target of ROS generated from both extrinsic and intrinsic sources, such as ultraviolet irradiation from the sun and metabolically-generated pro-oxidants, respectively. ROS increases c-Jun, a critical member of AP-1 complex, which in turn up-regulates CCN1 expression. With chronic exposure to ROS, elevated CCN1 impairs dermal fibroblasts production of type I collagen, and this impairment is a major contributing factor to thinning of the dermis and skin connective tissue aging. Antioxidant and dominant negative c-Jun mitigate ROS-induced skin connective tissue aging by suppressing CCN1. We previously reported that aging dermis has increased oxidative damage [5], c-Jun expression [50] and CCN1 expression [14], [17], suggesting ROS-driven connective tissue aging is mediated in part through the c-Jun/CCN1 axis. The present results support this hypothesis.

## Materials and Methods

### Ethics Statement

This study was conducted in compliance with Declaration of Helsinki principles. All procedures involving human subjects were approved by the University of Michigan Institutional Review Board, and informed written consent was obtained from all human subjects.

### Materials

Dulbecco’s Modified Eagle’s Media (DMEM), fetal calf sera, trypsin solution, and penicillin/streptomycin were purchased from Invitrogen (Carlsbad, CA). [γ-^32^P]ATP was obtained from New England Nuclear Life Science Products (Boston, MA). CCN1 antibody was purchased from Santa Cruz Biotechnology (Santa Cruz, CA). Type I collagen antibody was purchased from SouthernBiotech (Birmingham, AL). Total and phosphorylated c-Jun antibodies were purchased from Transduction Laboratories (San Diego, CA) and Santa Cruz Biotechnology (Santa Cruz, CA), respectively. β-actin antibody was purchased from Sigma Chemical (St. Louis, MO). All other reagents were purchased from Sigma Chemical.

### Procurement of human skin samples and ultraviolet irradiation

The study involving human volunteers was approved by the Institutional Review Board at the University of Michigan, and all subjects provided written informed consent before entering the study. Human skin punch biopsies were obtained from healthy adult human volunteers, as previously described [51]. N-acetyl-L-cysteine (20% NAC, American Regent Laboratories, Shirley NY) or its vehicle (70% ethanol) was applied to sun-protected buttock skin topically under occlusion for 24 hours before UV irradiation. Human skin was irradiated with 2MED (minimum erythema dose) solar-simulated UV (SPEC 450 W xenon arc solar simulator) [18], [49]. The UV dose that caused slight skin reddening (minimal erythema dose, MED) was determined for each subject 24 hours after UV irradiation. Full-thickness skin biopsies (4 mm) were taken 8 hours after UV exposure, in which UV irradiation maximally induces c-Jun and CCN1 [17], [18], [29]. Non-irradiated control skin were also taken at this time point.

### Cell culture, oxidative exposure, and antioxidant treatment

Human skin primary fibroblasts were established by outgrowth from human skin punch biopsies of healthy adult volunteers (20–55 years of age), as described previously [52]. Cells were maintained in DMEM supplemented with 10% fetal bovine serum, penicillin (100 U/ml), streptomycin (100 µg/ml) in a humidified incubator with 5% CO_2_ at 37°C. Cells were cultured at sub-confluence and utilized between passages 5 and 10. For oxidative exposure, cultured dermal fibroblasts were exposed to hydrogen peroxide (150 µM) in serum-free DMEM for one hour on two successive days. Cells were extensively washed after each exposure to remove the added hydrogen peroxide and placed in fresh DMEM containing 10% FBS. We found that this short-term exposure of dermal fibroblasts to low dose hydrogen peroxide on two successive days caused long-lasting elevation of intracellular ROS levels. Elevated intracellular ROS levels did not diminish for at least 21 days (the longest time point we have measured). We therefore used cells seven days after removing added hydrogen peroxide throughput all experiments. This protocol is referred to as ROS in this article. For some studies, dermal fibroblasts were treated with NAC (10 µM) for indicated times.

### RNA isolation and quantitative real-time RT-PCR

Total RNA was extracted from human skin punch biopsies and cultured dermal fibroblasts using a commercial kit (RNeasy mini kit, Qiagen, Chatsworth, CA) or TRIzol reagent (Invitrogen, Carlsbad, CA), respectively, according to the manufacturer’s protocol. For the PCR template, total RNA (200 ng) was reverse transcribed using Taqman Reverse Transcription kit (Applied Biosystems, Foster City, CA). Real-time polymerase chain reaction (PCR) was performed in duplicate with 2 µl of cDNA for all genes of interest and an internal control gene (36B4 housekeeping gene) using TaqMan Universal PCR Master Mix kit (Applied Biosystems, Foster City, CA) and a 7300 sequence detector system (Applied Biosystems). To ensure accuracy and reproducibility, PCR procedures were performed with a robotic workstation (Biomek 2000; Beckman Coulter, Inc., Hialeah, FL). PCR primers and probes were from Applied Biosystems custom oligonucleotide synthesis service. Primers for quantitative real-time PCR were described previously [15], [17]. Multiplex PCR reactions contained primers and probes for target gene and 36B4, a ribosomal protein used as an internal normalization control for quantitation. PCR amplification conditions were 35 cycles of: 94°C, 30 seconds; 54–60°C, 1 minute; 68°C, 1 minute; and 2°C, 10 minutes. Target gene expression levels were normalized to internal control gene.

### Western blot analysis

Whole cell extracts were prepared, and equal amounts of protein (∼50 µg/lane) were resolved by 12% sodium dodecyl sulfate-polyacrylamide (SDS) gel electrophoresis. The SDS gels were transferred to polyvinylidene difluoride membranes, and were blocked with PBST (0.1% Tween 20 in PBS) containing 5% nonfat milk for one hour at room temperature. Primary antibodies were incubated for one hour at room temperature, after which membranes were washed three times with PBST solution and incubated with appropriate secondary antibodies for one hour at room temperature. After washing three times with PBST, the membranes were developed with ECF (Vistra ECF Western blotting system, GE Health Care, Piscataway, NJ) following the manufacturer’s protocol. The membranes were scanned with a STORM PhosphorImager (Molecular Dynamics, Sunnyvale, CA), the intensities of each band were quantified using ImageQuant software (GE Health Care, Piscataway, NJ) and normalized to β-actin (control).

### Transfection and luciferase assay

CCN1 expression vector [14], dominant-negative mutant c-Jun (TAM67) [28], [29], and CCN1 siRNA (Quan et al. 2012) were transiently transfected by electroporation (Amaxa Nucleofector Koeln, Germany) according to the manufacturer’s protocol. After 48 hours of transfection, total RNA and cellular proteins were prepared, and mRNA and protein levels were determined by real-time RT-PCR and Western blot analysis, as described above. For CCN1 promoter/luciferase assay, the cells were transiently transfected with full-length CCN1 promoter/luciferase construct (−883/+1, CYR-900-luc), or AP-1 mutation construct (−883/+1, CYR-900APmut-luc), and AP-1 deletion construct (−605/+1, CYR-600-luc) as depicted in the Fig. 4a. These constructs were kindly provided Dr. Kunz, University of Rostock, Germany [27]. Cells were co-transfected with β-galactosidase expression vector as an internal control for transfection efficiency, as described previously [53]. Aliquots containing identical β-galactosidase activity were used for each luciferase assay. Luciferase activity was measured two days after transfection using an enhanced luciferase assay kit (PharMingen International, San Diego, CA) according to the manufacturer’s protocol.

### Measurement of ROS

Fibroblasts were cultured in DMEM containing 2,3,4,5,6-pentafluorodihydrotetra-methylrosamine (1 mmol/L RedoxSensor Red CC-1, Molecular Probes) for one hour. Oxidation of RedoxSensor Red CC-1, an indicator of levels of ROS, was visualized by fluorescence microscopy. Oxidation of RedoxSensor Red CC-1 was quantified by Image-pro Plus software (Media Cyberneyics, Rockville, MD). Briefly, the intensity of the red area corresponding to oxidized RedoxSensor Red CC-1-positive staining was measured from 10 random fields per slide. The software settings were programmed to quantify all nuclei (DAPI, blue staining) in the same fields, to yield the number of cells analyzed. Finally, the intensity of the red area (indication of ROS levels) was normalized by the member of cells and the results expressed as relative ROS level.

### Electrophoretic Mobility Shift Assay (EMSA), Statistical Analysis

EMSAs were performed as described previously [53] using nuclear extracts prepared using Nuclear and Cytoplasmic Extraction reagents (Pierce, Rockford, IL). Double-stranded oligonucleotide probes for EMSAs were as follows: CCN1 probe, 5′-AAATATTCCTGACTCAGAGACACAC-3′ (−842/−822, AP-1-binding site underlined); AP-1 consensus (5′-CGCTTGATGACTCAGCCGGAA-3′); and mutant (5′-CGCTTGATGACTTGGCCGGAA-3′) probes (mutations in AP-1-binding site underlined) (see Fig. 4b). CCN1 probe was synthesized from Invitrogen (Carlsbad, CA), and AP-1 consensus and mutant probes were purchased from Santa Cruz Biotechnology (Santa Cruz, CA). For competition experiments, a 50-fold molar excess of non-radioactive competitor probes was pre-incubated with the nuclear extract for 30 minutes on ice before adding [^32^P]-labeled probes. Gels were transferred to 3 MM Whatman paper, vacuum-dried, and scanned using the STORM PhosphorImager (Molecular Dynamics).

### Statistical Analysis

Data are expressed as mean ± SEM. Student's t-test was used to evaluate the statistical differences between the groups. All *p* values are two-tailed, and values less than 0.05 were considered statistically significant.

## Supporting Information

S1 Figure **CCN1 siRNA knockdown and it effects on basal levels of type I procollagen mRNA expression in human primary dermal fibroblasts.** Cells were transfected with control siRNA (CTRL si) or CCN1 siRNA (CCN1 si), and harvested two days later. (a) CCN1 mRNA levels were quantified by real-time RT-PCR and normalized to am internal control housekeeping gene (36B4). (b) CCN1 protein levels were determined by Western blot and normalized to an internal control (ß-actin). Inset shows representative blot. (c) Type I procollagen mRNA levels were quantified by real-time RT-PCR normalized to an internal control housekeeping gene (36B4). Data are means+SEM, N = 3, *p<0.05 vs. CTRL si.
(EPS)

S2 Figure **The effect of antioxidant treatment on the basal levels of CCN1 and type I procollagen mRNA in human primary dermal fibroblasts.** Cells were treated with vehicle (CRTL) or NAC (10 mM) for 24 hours. mRNA levels of CCN1 and type I procollagen were quantified by real-time RT-PCR and normalized to an internal control housekeeping gene (36B4). Data are means+SEM, N = 3.
(EPS)

S3 Figure **Effects of oxidative exposure on mRNA expression of CCN family members in human primary dermal fibroblasts.** Oxidative exposed dermal fibroblasts were prepared as described in Methods. mRNA levels of CCN family members were quantified by real-time RT-PCR and normalized to an internal control housekeeping gene (36B4). PCR primers for quantitative real-time PCR were described previously (15). Data are means+SEM, N = 3, *p<0.05 vs. CTRL.
(EPS)
